# The Role of Deubiquitinating Enzymes in Acute Lung Injury and Acute Respiratory Distress Syndrome

**DOI:** 10.3390/ijms21144842

**Published:** 2020-07-08

**Authors:** Tiao Li, Chunbin Zou

**Affiliations:** Division of Pulmonary, Allergy, Critical Care Medicine, Department of Medicine, University of Pittsburgh School of Medicine, Pittsburgh, PA 15213, USA; tiaoli@pitt.edu

**Keywords:** acute lung injury/acute respiratory distress syndrome, deubiquitinating enzyme, protein stability, inflammation, infection

## Abstract

Acute lung injury and acute respiratory distress syndrome (ALI/ARDS) are characterized by an inflammatory response, alveolar edema, and hypoxemia. ARDS occurs most often in the settings of pneumonia, sepsis, aspiration of gastric contents, or severe trauma. The prevalence of ARDS is approximately 10% in patients of intensive care. There is no effective remedy with mortality high at 30–40%. Most functional proteins are dynamic and stringently governed by ubiquitin proteasomal degradation. Protein ubiquitination is reversible, the covalently attached monoubiquitin or polyubiquitin moieties within the targeted protein can be removed by a group of enzymes called deubiquitinating enzymes (DUBs). Deubiquitination plays an important role in the pathobiology of ALI/ARDS as it regulates proteins critical in engagement of the alveolo-capillary barrier and in the inflammatory response. In this review, we provide an overview of how DUBs emerge in pathogen-induced pulmonary inflammation and related aspects in ALI/ARDS. Better understanding of deubiquitination-relatedsignaling may lead to novel therapeutic approaches by targeting specific elements of the deubiquitination pathways.

## 1. Introduction

Acute lung injury and acute respiratory distress syndrome (ALI/ARDS) are a group of illnesses with features of lung inflammation, air–blood barrier disfunction, and hypoxemia. ALI/ARDS are life-threatening with a severe public health concern, approximately 200,000 people per year develop into ALI/ARDS in the United States, and the mortality rates are high at 30–40% [1,2,3,4,5]. It is believed that about ~10% of patients in intensive care units eventually develop into ALI/ARDS worldwide. Etiologically, microbial pneumonia, sepsis, aspiration of gastric contents, or severe trauma are the major causes of ALI/ARDS. Approximately 40% of the ALI/ARDS patients are linked with viral and bacterial pneumonia. The outbreak of severe acute respiratory syndrome coronavirus 2 (SARS-CoV-2), which causes coronavirus disease 2019 (COVID-19), has become a pandemic disease. By now, millions of people have suffered from this disease with hundreds of thousands of deaths in almost all countries all over the world because of the pandemic, and the numbers of the diagnosed patients and the deaths due to this disease are climbing each day (https://coronavirus.jhu.edu/map). For severe COVID-19 patients, ALI/ARDS represent one of the major pathological changes; phenotypes include inflammatory infiltration and inflammatory storm, alveolar epithelial–capillary damage, lung embolism and hemorrhage, hypoxia, and poor prognosis with high mortality. The pathobiology of the disease is incompletely understood [1,6,7,8]. Furthermore, no specific effective therapeutic method has been developed to treat the illness. Thus, understanding the molecular mechanisms of ALI/ARDS is of particular important in developing effective remedies against the illness.

Overwhelmed immune responses are believed to be a major contributing factor in the pathogenesis of ALI/ARDS. In the initial pulmonary infection, invaded microbial pathogens including viruses and bacteria attract and activate residential microphages to release chemokines and cytokines, along with infiltration of leukocytes, particularly neutrophils and lymphocytes, into the alveolar sacs [5,9]. In ideal scenarios, host immune responses clear and exclude the invaded pathogens and repair the diseased tissues. However, host immune responses may be unable to achieve this goal due to the pathogenicity of the microbe or the compromised capacity of the host defense, such as in patients with cancer, organ transplantation, diabetes, or HIV infection. Higher inflammatory responses may occur in these cases and an over-reacted inflammatory response eventually leads to an overwhelmed inflammatory response. An overwhelmed inflammatory response is increasingly noticed as one of the key contributors to the poor prognosis of ALI/ARDS. A dysregulated high inflammatory response, also referred to as a “cytokine storm”, increases mortality in ALI/ARDS patients [10,11]. Along with the process of cytokine storm, dysregulated molecular signaling may cause deleterious damage independent of microbial pathogens that increase mortality. However, a high inflammatory storm turns into low inflammation in the later stage due to immune paralysis that may lead to immunosuppression, which contributes to secondary infection and worsens the prognosis of the patients as well [1].

In the meantime, the invasion of microbial pathogens causes airway epithelial and pulmonary endothelial cell death, destroys alveolar architecture, and damages the air–blood barrier [5,12]. These pathological changes impair effective air–blood exchange, which results in edema and hypoxemia. Clinically, hypoxemia in patients with ARDS is caused by ventilation-to-perfusion mismatch, as well as right-to-left intrapulmonary shunting [4]. In addition, impaired excretion of carbon dioxide is a major component of respiratory failure, resulting in elevated minute ventilation that is associated with an increase in pulmonary dead space (that is, the volume of a breath that does not participate in carbon dioxide excretion). Elevation of pulmonary dead space and a decrease in respiratory compliance are independent predictors of mortality in ARDS [13]. The pathophysiological mechanisms of ALI/ARDS are yet to be fully understood. A large number of signal transduction pathways have been revealed to be involved in this process. Signal transductions in control of protein stability and availability, including protein ubiquitination and degradation, are typical among the pathways. Several review articles have introduced the role of ubiquitination and proteolysis in lung diseases [14,15,16]. In this review, we summarize recent findings regarding the importance of deubiquitination and DUBs in regulation of inflammation and related pathologies and highlight the role of DUBs in ALI/ARDS.

## 2. Protein Ubiquitin Proteasomal Degradation and Deubiquitination

**Figure 1:**
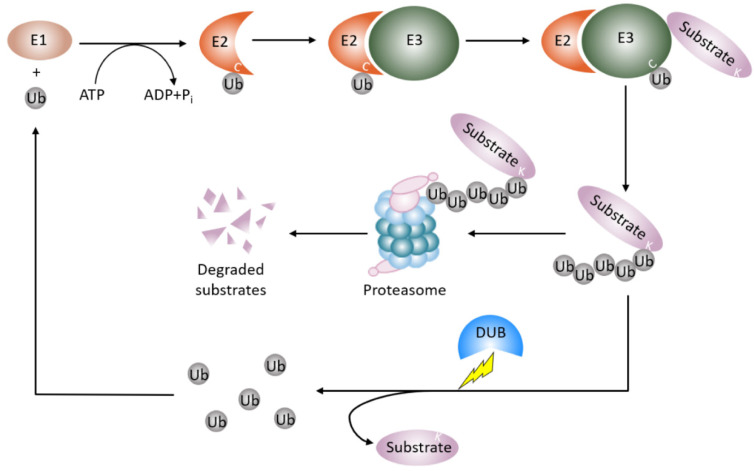
Protein ubiquitin proteasomal degradation and deubiquitination. A protein destined for degradation unleashes a cascade of enzymatic activity involving ubiquitination and proteasomal degradation. E1 Ub-activating enzymes activate ubiquitin and pass the ubiquitin to E2-Ub-conjugating enzymes. E3 Ub-ligases recognize the protein substrates and couple E2-Ub-conjugating enzymes to covalently add the ubiquitin or ubiquitin moieties to the protein substrates. The ubiquitinated proteins are then degraded by the proteasome. Deubiquitinating enzymes remove the mono-ubiquitin or polyubiquitin chains from the ubiquitinated protein to stabilize the protein from proteasomal degradation and recycle ubiquitin units. Ub: ubiquitin; E1: E1 Ub-activating enzyme; E2: E2-Ub-conjugating enzyme, E3: E3 Ub-ligases; DUB: deubiquitinating enzyme.

Proteins dynamically exert their diversified functions in life processes in response to different pathophysiological settings. In concert with gene transcription, ubiquitin proteasome degradation governs the abundance and availability of the protein in the cell. Most of the proteins modified by a post translational modification called ubiquitination are deemed to be degraded [17,18]. Ubiquitination involves the covalent attachment of the small conserved protein called ubiquitin (Ub, 87 amino acids in length) to a target protein, almost exclusively at a lysine residue. Ubiquitination is an enzymatic cascade that requires the orchestrated interplay of three different enzymes (Figure 1). E1 Ub-activating enzymes bind to both ATP and ubiquitin and expose a cysteine residue, the active site of ubiquitin, with the release of an AMP. E2 Ub-conjugating enzymes take over activated ubiquitin from E1 enzymes and cooperate with E3 Ub-ligases. The E3 Ub-ligases interact with E2 enzymes and recruit protein substrates to initiate conjugation of single ubiquitin or polymeric ubiquitin chains to the protein substrates. E3 Ub-ligases recognize the protein substrates and determine the specificity of protein substrates [19,20]. In humans, there are two E1 Ub-activating enzymes, 14 E2 Ub-conjugating enzymes, and approximately 1000 E3 Ub-ligases [21].

The process of ubiquitination is reversible, a group of enzymes called deubiquitination enzymes conduct the enzymatic process [22] (Figure 1). Deubiquitination is the reverse process of ubiquitination, that removes the mono-ubiquitin and poly-ubiquitin chains from the modified proteins to generate free ubiquitin, which terminates the function of ubiquitinated protein, and specifically, stabilizes the ubiquitinated protein from degradation. Deubiquitination also replenishes the ubiquitin pool, and maintains homeostasis of the cellular ubiquitin [23]. This process is performed by deubiquitinating enzymes (DUBs), which are a large set of proteases. The number of DUBs in humans is about 100, while ~20 DUBs exist in the yeast *Saccharomyces cerevisiae* [24,25,26]. A number of approaches are utilized in studying DUBs and the related diseases. These approaches include conventional protein–protein interaction techniques such as immunoprecipitation, enzymatic assays, bioinformatics, proteomic, transcriptomic, and structure analysis techniques. Based on the architecture of their catalytic domains, to date, six structurally distinct DUB families have been described [27]. Five families of DUBs are cysteine proteases, including54 members of USPs(ubiquitin-specific proteases)in humans, four members of UCHs(ubiquitin carboxy-terminal hydrolases), 16 members of OTUs(ovarian tumor proteases), four members of MJDs (Machado–Josephin disease protein domain protease) [25,26], and four members of MINDYs (motif interacting with ubiquitin (MIU)-containing novel DUB family) [28]. The sixth subfamily is JAMMs (Zn-JAB1/MPN/MOV34 domain protease), which includes a conserved zinc metallopeptidase [25,26]. All DUB family members bear a catalytic domain that removes ubiquitin from the protein substrates [27]. The catalytic domain of MIU family sub-members is a new folding variant within the superfamily of cysteine protease and shows a remarkable selectivity for cleaving long lysine 48 (K48)-linked ubiquitin chains. In particular, cleavage selectivity of DUBs is determined by catalytic domain alone, whereas a DUB called MINDY requires a motif interacting with ubiquitin (MIU) as well as a catalytic domain for maximal DUB activity [28]. The physiological roles of DUBs include controlling protein stability and quality, maintaining ubiquitin homeostasis, and regulating ubiquitin signals against the functions of E3 Ub ligase [23]. Therefore, DUBs regulate numerous cellular events such as the cell cycle, DNA damage response, inflammatory signaling, and proliferation and cell death.

## 3. Molecular Mechanisms of DUBs in the Pathogenesis of ALI/ARDS

Mounting studies have focused on inflammation to dissect its underlying molecular mechanisms in the pathogenesis of ALI/ARDS. Deubiquitinating enzymes play crucial roles in modulation of inflammation by changing the protein stability of the critical molecules (Table 1). Several USPs have proved to play emerging roles in the regulation of lung inflammation [29,30]. Innate immunity provides the first line of host defense against pathogens. In lung inflammation, USP14 protein is over-expressed, reducing I-κB protein levels and thus increasing cytokine release in lung epithelial cells [31,32]. USP7 acts as a negative regulator of the NF-κB pathway by mediating the deubiquitination of NEMO, TRAF6 and IKKγ, which leads to the retention of NF-κB in the cytosol, thus suppressing its activity [33,34]. Pro and anti-inflammatory cytokines increase in bronchoalveolar lavage fluid and circulating plasma of patients at different stages of ARDS. TNF-α and IL-1β are important proinflammatory cytokines in the pathogenesis of ARDS [35]. After their receptor activation, cIAP-mediated K63-ubiquitination of RIPK1 and the TRAF proteins leads to the recruitment of linear ubiquitin chain assembly complex (LUBAC). The stability of lysophosphatidic acid receptor 1 (LPA1) is up-regulated by ubiquitin-specific protease 11 (USP11), which deubiquitinates LPA1 and enhances LPA1-mediated proinflammatory effects [33,36,37,38,39]. Furthermore, the deubiquitinating enzyme USP13 stabilizes the anti-inflammatory receptor IL-1R8/Sigirr to suppress lung inflammation [40,41,42].

Alveolar residential macrophages are central to the development of the inflammatory response by recruiting neutrophils and circulating macrophages to the site of injury, their functions are modulated by deubiquitinating enzymes [96,97]. These cells secrete cytokines, chemokines, reactive oxygen species, proteases, and other mediators that modulate the inflammatory responses and injure the alveolocapillary barrier. Gram-negative bacteria-derived endotoxin lipopolysaccharide (LPS) promotes stability of a histone acetyltransferase HBO1 via the function of USP25. HBO1 is believed to fire DNA replication licensing at the S-phase of the cell cycle, however, it also regulates inflammatory gene transcription in settings of pulmonary infection. USP25-stabilized HBO1 promotes inflammatory gene transcription in monocyte THP-1 cells [67]. In addition, inhibition of USP7 and USP47 blocks the NLRP3 inflammasome by preventing apeck-like protein containing a CARD (ASC) oligomerization and speck formation in macrophages [38]. USP17 mediates macrophage-promoted inflammation and stemness in lung cancer cells by regulating TRAF2/TRAF3 complex formation [59]. The activity of deubiquitination regulates inflammasome assembly and function. Deubiquitination of NLRP3 has been suggested to contribute to inflammasome activation. Upon treatment with NLRP3 ligands after the priming step, ABRO1, a subunit of the BRISC deubiquitinating complex, is required for optimal NLRP3-ASC complex formation, ASC oligomerization, caspase-1 activation, and IL-1β and IL-18 production. This evidence indicates that efficient NLRP3 activation requires ABRO1 [98]. Protein kinase JNK1 catalyzes NLRP3 phosphorylation at S194 within NLRP3, which is critical for NLRP3 deubiquitination and facilitates its self-association and the subsequent inflammasome assembly [99]. Another inflammasome component NALP7 is regulated by the deubiquitinating enzyme STAM-binding protein (STAMBP), targeting the STAMBP with a small molecule that inhibits NALP7 inflammasome activity [95].

The activities of deubiquitinating enzymes are involved in many aspects of the pathogenesis in ALI/ARD. Lung epithelial cell death is a hallmark in ALI/ARDS. Massive lung epithelial cell death has been reported in ARDS patients. Lung epithelial cell death is regulated by deubiquitinating enzymes. Loss of DUB CYLD can activate NF-κB to inhibit apoptosis in lung epithelial cells [100]. In lung infection, USP13 are aberrantly expressed, inhibition of USP13 reduces the abundance of anti-apoptotic protein MCL1 in the lung [42]. On the other hand, recent mechanistic studies have reported that lung epithelial cells may defend from bacterial invasion through several mechanisms. USP25 may regulate the degradation of a deacetylation enzyme HDAC11 to modulate cellular *Pseudomonas aeruginosa* bacterial load, probably via interferon signaling in bronchial lung epithelial cells [68]. OTUB1 interferes with bacterial uptake by modulating the RhoA level [78]. Furthermore, deubiquitination has been proposed to play an important role in alveolar epithelial dysfunction during ALI. USP10 exerts an effect on mucociliary clearance by regulating the endocytic recycling of the cystic fibrosis transmembrane conductance regulator (CFTR) in airway epithelial cells [37,52]. In addition, accumulating data suggest that deubiquitination may regulate structural components of the alveolar epithelial monolayer. Structural integrity of epithelial cells and intercellular junctions plays an important role in the maintenance of alveolar epithelial barrier integrity. A study suggests that phosphorylated E2F1 is stabilized by nuclear USP11 to drive Peg10 gene expression and activate proliferation of lung epithelial cells [54]. Finally, airway barrier integrity is primarily maintained by intercellular junctions, which in turn control the paracellular transport of proteins, fluids, and small molecules. Cell junction and junctional protein recycling and remodeling is pivotal in barrier integrity. Deubiquitination and DUBs have been shown to regulate adherence of junctional proteins [101]. For example, USP48 regulates E-cadherin mRNA levels through stabilizing the TRAF2-JNK pathway in lung epithelial cells [69]. This study exhibits an indirect effect of DUBs on regulation of E-cadherin levels and lung epithelial barrier integrity.

Until now, the mechanism of COVID-19 infection has not been well illustrated yet. From the biopsy or autopsy of COVID-19 patients, diffuse damage of lung parenchyma has been shown [102,103]. Experts hypothesized that SARS-COV-2 invasion severely interrupts the integrity of the airway barrier, thus inducing aberrant inflammatory release (“cytokine storm”) and further worsening the lung injury and microcirculation dysfunction, resulting in uncontrolled sepsis in severe cases [104]. Whether DUBs participate in the mechanism of SARS-COV-2 infection has not been reported. The coronavirus family contains six members. SARS-CoV and Middle East respiratory syndrome coronavirus (MERS-CoV) are the two members that have brought an epidemic in recent years. SARS-CoV and MERS-CoV, containing the papain-like cysteine proteases (PLpro), termed SARS-CoVPLpro and MERS-CoVPLpro respectively, are antagonists of the host antiviral immune response as they remove ubiquitin and its modifier interferon-stimulated gene 15 (ISG15) signals from host cell factors [105,106]. Whether such a protease encoded by the SARS-CoV-2 genome exists has not been reported, which might expand the field of SARS-CoV-2 study. Furthermore, human DUBs might be potential targets for SARS-CoV-2 invasion. We scanned the related dataset of genes and proteins in COVID-19 and the SARS-CoV-2 infected cells. Data showed that a majority of DUBs are decreased in human iPSC-cardiomyocytes infected with SARS-COV-2 via RNA-sequencing [107]. In ACE2 positive type II pneumocytes, a number of USPs including USP11 and USP38 are elevated compared to ACE2 negative cells using next generation sequencing [108]. SARS-CoV-2 spike (S) protein invades human tissue through binding angiotensin-converting enzyme 2 (ACE2), which reminds us that USPs might play an important role in COVID-19 development. However, in the sera of COVID-19 patients, no DUBs have been found through proteomics [109]. In all, the above data revealed that DUBs might be involved in the mechanism of SARS-CoV-2 infections, but further studies are still urged to explore the function of DUBs in COVID-19.

## 4. Deubiquitinating Enzymes Involved in ALI/ARDS

### 4.1. USPs

The USP subfamily contains the majority of DUBs encoded by the human genome, which are the most diversified members within the DUB family [110,111,112]. The most studied DUB family member in USPs is cylindromatosis (CYLD). CYLD was originally identified as a tumor suppressor, where loss of which causes a benign human syndrome CYLD [113]. With sequence homology to the catalytic domain of ubiquitin carboxy-terminal hydrolases (UCH), CYLD cleaves K63-linked polyubiquitin chains off its target proteins [114,115,116]. CYLD is proven to be induced by Gram-negative and Gram-positive bacterial pathogens or their products [45,46,48,117]. The transcription factor NF-κB activated by bacteria is essential for induction of CYLD, in turn, induced CYLD negatively regulates the bacteria induced NF-κB signaling [46,117]. CYLD deubiquitinates TRAF6 and TRAF7 to negatively regulate peptidoglycan-induced Toll-Like receptor 2 (TLR2) signaling and inflammation [45]. CYLD is also highly induced by pneumolysin (PLY). CYLD deficiency protects mice from acute lung injury in lethal *Streptococcus pneumoniae* infections by inhibiting plasminogen activator inhibitor-1 (PAI-1) expression [44,48]. Furthermore, evidence shows that CYLD negatively regulates the *S. pneumoniae*-induced nuclear factor of activated T cells (NFAT)signaling pathway by deubiquitinating TGF-β-activated kinase 1(TAK1) [43]. In contrast, CYLD(-/-) mice are hypersusceptible to *Escherichia coli* pneumonia with enhanced NF-κB activation [118]. Perhaps different pathogens may use distinct mechanisms to promote lung inflammation. In the late stage of bacterial infection, CYLD exhibits negative effects on injury-induced lung fibrotic response by inhibiting TGF-β-signaling [47]. These discoveries indicated that CYLD might possess a potential drug target for the treatment of bacterial infection pneumonia.

USP7 (HAUSP)is originally identified as a viral binding protein that preferentially cleaves K11-, K63- and K48-linked ubiquitin chains [119,120]. USP7 is involved in viral infection by targeting virus related protein to modulate virus replication and production [49,50,51]. USP7 is reported to deubiquitinate and stabilize NF-κB to increase its transcriptional activity in TLR-induced inflammatory gene expression [39]. Furthermore, USP10 fine-tunes NOTCH signaling in angiogenic sprouting by deubiquitinatingNOTCH1 intracellular domain (NICD1) to slow down its turnover of the short-lived form of the activated NOTCH1 receptor [53].

UPS13 is also reported to regulate antiviral responses, however, its function is controversial. USP13 is considered to promote IFN signaling and play an antiviral role by stabilizing STAT1 [55]. Nevertheless, USP13 deficiency enhances antiviral responses through deubiquitinating stimulator of interferon (STING) [56]. During bacterial infection, USP15 loses its activity for IκBα deubiquitination by interacting with E3 Ub-ligase Hrd1 to promote TLR4-induced inflammation [57]. USP17 mediates deubiquitination and stabilization of HDAC2 in cigarette smoke extract-induced inflammation [58]. USP19 also preserves a negative effect on TNF-α- and IL-1β-triggered NF-κB activation by deubiquitinating TAK1 [60]. USP19 interacts with TIR domain-containing adaptor inducing interferon-β (TRIF), and thus impairs its recruitment to TLR3/4 [61]. USP19 deficient mice produce exacerbated inflammatory cytokines and are more susceptible to septicemia death [60,61]. USP19 affects DDX58/RIG-I-mediated type I interferon signaling through ubiquitinating BECN1 and promoting the formation of autophagosomes [62]. USP25 plays a protective role in virus or bacterial infection. Several studies showed that USP25 negatively regulates virus-induced type I IFN signaling by stabilizing TRAF2, TRAF3 and TRAF6 [64,65,75,87,121,122,123,124]. Furthermore, USP25 inhibits TLR4-activated innate immunity via removing K48 ubiquitination of TRAF3 [63]. USP25 deficient mice have been shown to be more susceptible to virus infection and LPS-induced septic shock [63,65].IL-17-mediated inflammation is also attenuated by USP25 through TRAF5 and TRAF6 deubiquitination [66]. The anti-malarial drug chloroquine is suggested to alleviate LPS-induced inflammation by up regulatingUSP25 in macrophages [125].

### 4.2. OTUs

The OTU family DUBs can be divided into four subfamilies, including OTULINs (OTULIN and FAM105A), OTUBs/Otubains (OTUB1 and OTUB2), OTUDs (OTUD1, OTUD2/YOD1, OTUD3, OTUD4, OTUD5/DUBA, OTUD6A, OTUD6B, ALG13, and HIN1L), and A20s (A20, Cezanne, Cezanne2, TRABID, and VCPIP) [126]. The majority of OTU members are reported to regulate pathogen-induced cell signaling cascades. In innate and adaptive immunity, OTULIN is an essential negative regulator of LUBAC, which hydrolyzes LUBAC induced Met-1 lineal ubiquitination to prevent NF-κB- or TNF-induced inflammation augmentation [71,72,73,127]. OTULIN can also control antiviral signaling by regulating the lineal ubiquitination chain of STAT1 [74]. For the negative role of OTULIN in immune responses, OTULIN deficiency might cause auto-inflammatory syndrome [128].

OTUB1 and OTUB2 regulate virus-triggered IFN inflammation by deubiquitinating TRAF3 and TRAF6 [75]. OTUB1 suppresses the E3 ubiquitin-ligase by co-opting K48 ubiquitin recognition to regulate DNA damage [76,129,130,131,132]. Recent studies also show that OTUB1 augments NF-κB-dependent immune responses in dendritic cells in infection and inflammation by stabilizing UBC13 [76]. OTUB1 recruits phosphorylated SMAD2/3 and inhibits its ubiquitination by binding with E2 Ub-conjugating enzyme to enhance TGF-β signaling [80]. OTUB1 regulates the maturation and activation of NK and CD8+T cells via inhibiting AKT ubiquitination [77]. Furthermore, virus-induced OTUB1 degradation blocks the RIG-I-dependent immune signaling cascade and antiviral response [79]. Several studies showed that OTUD1 plays an important role in inflammation regulation [81,82]. RNA viruses induceOTUD1 to promote the degradation of the MAVS/TRAF3/TRAF6 signalosome to inhibit innate immunity [81]. Furthermore, OTUD1 inhibits type 1 IFN induction after virus infection through cleaving noncanonical K6-linked ubiquitination of IRF3 [83].

OTUD1 knockout mice show more resistance to virus infection and LPS stimulation [81,83]. OTUD4 is a K48-specific deubiquitinating enzyme that is previously been reported to maintain the stability of the alkylation repair enzyme ALKBH3 for promoting DNA damage repair [86]. However, OTUD4 also preserves K63-linked deubiquitinating activity, specifically targetingMyD88 to inhibit NF-κB signaling [84]. A recent study shows the role of OTUD4 in innate antiviral immunity. OTUD4 is induced by virus infection and targets MAVS ubiquitination, triggeringIRF3 and NF-κB signaling to sustain antiviral responses [85].Like most of the DUBs, the family member A20 shows the negative effect on the activation of NF-κB signaling [87,88,133].Myeloid-A20-deficiency shows a higher inflammatory reaction and sustained NF-κB activation [133]. A20 terminates TLR signals by targeting TRAF6 deubiquitination [87]. Similar to OTUB1, A20 suppresses NF-κB signaling by conjugating to E3 Ub-ligase [88]. Histone methyltransferase-enhanced A20 can also suppress the inflammatory response by modulation of NEMO and deubiquitination of TRAF6 [134]. Due to its role in inflammation inhibition, A20 induced by TNFα participates in age-related macrophage dysfunction in the lung [135]. OTUDs are newly discovered in antiviral immune responses, which reminds us of the potential drug target for the treatment of virus-induced lung injury.

### 4.3. JAMMs

The JAMMs are the third largest subfamily in DUBs, and it comprises 12 members: COP9 signalosome subunit (CSN)5, 26S proteasome non-ATPase regulatory subunit 14 (POH1), BRCA1/BRCA2-containing complex subunit 3 (BRCC3, also known as BRCC36 in humans), MPN domain containing (MPND, myb-like SWIRM and MPN domains 1 (MYSM1), eukaryotic translation initiation factor 3 subunit (EIF3)H, CSN6,26S proteasome non-ATPase regulatory subunit 7 (PSMD7), EIF3F, anti-Müllerian hormone (AMSH), AMSH-LP, and pre-mRNA-processing-splicing factor 8 (PRPF8) [93,136,137,138,139].STAMBP (also known as the associated molecule with the SH3 domain of STAM or AMSH), a metalloprotease and a member of the Jab1/MPN metalloenzyme (JAMM) family of DUBs, impedes the lysosomal degradation of NACHT, LRR and PYD domain-containing protein 7 (NALP7)to inhibit inflammasome activity [95,140]. POH1 deubiquitinates pro-IL-1β and inhibits mature IL-1β production, thus restricting inflammasome activity and LPS-induced inflammation [93]. DUBBRCC3 forms a multi-protein complex (BRISC) with ABRO1, NBA1, and BRE that specifically cleaves K63-linked ubiquitin in the cytoplasm [141]. ABRO1 is important in efficient NLRP3 activation. ABRO1 deubiquitinates NLRP3 to promote NLRP3 inflammasome activation [98]. BRCC3 also targets NLRP2 to regulate inflammasome formation [94].

### 4.4. OTHER DUBs

The enzymes of the UCH protein family includes four members, UCHL1/PGP9.5 (protein gene product 9.5), UCHL3, UCHL5/UCH37, and BRCA1 associated protein-1(BAP1), which contain a conserved catalytic UCH domain of ~230 amino acids [142,143]. The activities of these proteins have been associated with the occurrence and development of cancer [143]. UCHL5/UCH37 is suggested to play an anti-apoptotic role in lung epithelial cells through altering Bax/Bcl-2, caspase 3, and caspase9 signals [144]. UCH5/UCH37 deubiquitinates both smad2 and smad3 to promote TGFβ-1 induced lung fibrosis [70]. Studies of UCHs in lung injury and pathogen invasion are still lacking.

The MJD family onlycontains four members: Ataxin (ATXN)3, ATXN3L, Josephin domain containing (JOSD), and JOSD2 [145]. Studies show that ATXN3 andJOSD1 are involved in antiviral responses. ATXN3 enhances type 1 IFN signaling during viral infection through deubiquitinating and stabilizing HDAC3 [90]. Nevertheless, JOSD1 exhibits a negative role in antiviral activity. JOSD1 inhibits the IFN signal cascade via deubiquitinating and stabilizing SOCS1 [92].

The MCPIP, also known as ZC3H12A (zinc finger CCCH-type containing 12A) family includes MCPIP1-7 members [146,147,148]. MCPIP implicates a negative role in regulation of the cellular inflammatory responses [149]. MCPIP1 is the most studied in the MCPIP family. Acting as a deubiquitinating enzyme, MCPIP1 inhibits NF-κB and c-Jun N-terminal kinase (JNK) signaling pathways by removing the ubiquitin moieties from TNF receptor-associated factors (TRAFs), including TRAF2, TRAF3 and TRAF6 [150].As an RNase, MCPIP also regulates inflammatory cytokines like IL-6 by regulating RNA decay [151] and innate defense via degrading viral RNA [152]. MCPIP deubiquitinates TRAF6 to impede NF-κB signaling [89].

Like the recently identified DUBs, the MINDY family contains four members: MINDY1–4 [145], which are highly selective at hydrolyzing K48-linked poly-ubiquitin. No data about MINDYs in ALI/ARDS pathogenesis has been reported. The above DUBs play essential roles in the initial development of cancer. However, their functions in lung injury are not fully elucidated.

## 5. Potential Therapeutic Approaches Targeting DUBS in ALI/ARDS

Pathogen-related DUBs are promising potential targets of drug discovery for human pathogen infection and associated inflammatory disorders. Bacteria-encoded DUBs might promote bacterial pathogenicity through inhibiting the human ubiquitin–proteasome system [153]. Furthermore, viruses with genes for DUBs might inhibit the antiviral pathways using a DUB strategy to modulate protein–protein interactions. The SARS-CoVPLpro and MERS-CoVPLpro papain-like cysteine proteases have been reported, showing a conserved similar structure to the USP family of DUBs by X-ray structure, which shows the potential targets of DUBs for antiviral drug discovery [154,155]. In addition, several RNA virus-related proteases containing the OUT domain can also remove ubiquitin and ISG-15 signals from host cellular proteins, which represents a potential promising domain for antiviral therapy [156]. The above findings present great interest to explore a DUB-associated anti-infective strategy for human pathogen invasions. However, despite the possibility of DUBs as drug targets, the drug discovery for ALI/ARDS is still challenging, with few DUB inhibitors or activators having been explored.

## 6. Conclusions and Future Perspectives

**Figure 2:**
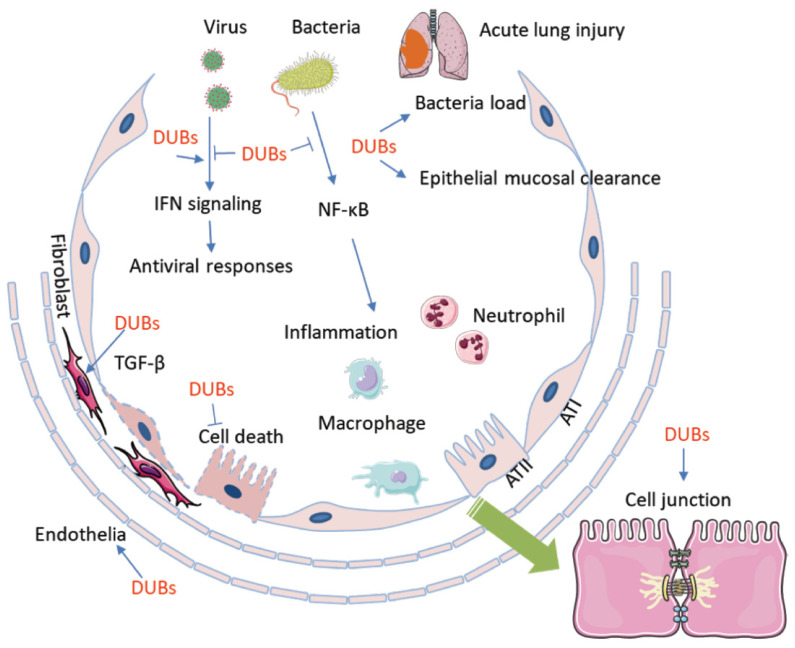
Deubiquitination and DUBs are involved in the pathogenesis of ALI/ARDS. DUBs conduct deubiquitination that is exclusively involved in every aspects of the pathogenesis of ALI/ARDS. Microbial pathogens regulate the activity and availability of DUBs to impact host immune defense and the inflammatory response, which includes chemokine and cytokine release, macrophage activation, and neutrophil and lymphocyte infiltration. On the other hand, DUBs participate in pathogen-mediated lung epithelial and endothelial cell proliferation and death. Furthermore, DUBs may affect epithelial mucosal clearance and regulate the bacterial load in small airway alveolar epithelial cells. In addition, DUBs impair cell junctions and the air–blood barrier. AT1: alveolar type 1 epithelial cell; AT2: alvelolar type 2 epithelial cell; TGF-β: transforming growth factor-β; DUB: Deubiquinating enzyme.

During the past decade, studies began to dissect the role of DUBs in ALI/ARDS. Increasing evidence proved that immune responses, inflammation, cell death, air–blood barrier integrity, and invasiveness of the pathogens are fine-tuned by DUBs in ALI/ARDS (Figure 2). Modulation of critical proteins via UPS and DUBs plays a central role in the pathogenesis of diseases such as cancer and autoimmune disease. Furthermore, DUBs are drawing increasing interest as therapeutic targets against these diseases. Our understanding of DUBs in ALI/ARDS is limited, and the specific role of DUBs remains largely unknown. Particularly, the global outbreak of COVID-19 has raised the demand for research on the pathological mechanisms of ALI/ARDS. Discovery of the role of DUBs in ALI/ARDS might bring valuable information on the pathogenesis of the illness and thereafter drug discovery. The diversified microbial pathogens may cause ALI/ARDS via distinct molecular mechanisms, which increase the complexity of the whole picture that we are attempting to figure out. On the other hand, the current studies are mostly focused on the function of DUBs on the regulation of protein degradation and stability. The functions of DUBs other than protein stability are yet to be studied in the setting of ALI/ARDS.As a post-translational modification, ubiquitinated proteins may exert a range of functions in life processes and in the pathogenesis of ALI/ARDS, such as signaling transduced via ubiquitinated protein. It is hoped that more data on DUBs might lead to identification of novel molecular mechanisms in ALI/ARDS, thus allowing the development of specific DUB inhibitors/agonists for the treatment of this acute and severe respiratory illness.

## Figures and Tables

**Table 1:** The roles of DUBs in ALI/ARDS.

DUBs	Target Genes	Function
CYLD	TAK1 [43]	Negatively regulates *S.p*. induced NFAT signaling [43]
	TRAF6 [44]	Inhibits *S.p*. induced PAI-1 expression [44]
	TRAF6/TRAF7 [45,46]	Regulates TLR4 signaling [45] Inhibits inflammation [46]
	AKT [47]	Regulates TGF- β signaling [47]
	PAI-1 [48]	Regulates acute lung injury [48]
USP-7	NLRP3 [38]	Regulates NLRP3 inflammasome activation [38]
	NF-κB [39], NEMO [33]	Regulates NF-κB signaling [33,39]
	VP24 [49]	Involves in virus replication [49]
	Tat [50]	Involves in virus production [50]
	TRAF3/TRAF6 [51]	Modulates antiviral signaling [51]
	TRAF6/IKKγ [34]	Regulates TLR signaling [34]
USP-10	CFTR [37,52]	Epithelial mucosal clearance [37,52]
	NICD1 [53]	Regulates Notch signaling [53]
USP-11	E2F1 [54]	Regulates lung epithelia proliferation and wound healing [54]
	LPA1 [36]	Enhances inflammation [36]
USP-13	IL-1R8/Sigirr [40]	Suppresses lung inflammation [40]
	PTEN [41]	Regulates cell apoptosis [41]
	MCL1 [42]	Regulates transformation of fibroblasts [42]
	STAT1 [55]	Regulates IFN Signaling [55]
	STING [56]	Negatively regulates antiviral responses [56]
USP-14	I-kB [31]	Increases cytokine release [31]
	CBP [32]	Lung inflammation [32]
USP-15	IκBα [57]	NF-κB activation [57]
USP-17	HDAC2 [58]	Reverses glucocorticoid resistance [58]
	TRAF2/TRAF3 [59]	Lung inflammation [59]
USP-19	TAK1 [60]	Inhibits NF-κB activation [60]
	TRIF [61]	Inactivates TLR3/4-mediated innate immune responses [61]
	BECN1 [62]	Promotes formation of autophagosomes and inhibits DDX58/RIG-I-mediated type I interferon signaling [62]
USP-25	TRAF3 [63]	Regulates TLR4-dependent Innate Immune Responses [63]
	RIG-I/TRAF2/TRAF6 [64]	Negatively regulates virus-induced type I interferon signaling [64]
	TRAF3/TRAF6 [65]	Promotes Innate Antiviral Responses [65]
	TRAF5 and TRAF6 [66]	Regulates IL-17 signaling [66]
	HBO1 [67]	Modulates macrophage inflammation [67]
	HDAC11 [68]	Modulates bacteria load [68]
USP-48	TRAF2 [69]	Reduces E-cadherin-mediated adherence junctions [69]
UCHL5(UCH37)	Smad2/Smad3 [70]	Promotes TGFβ-1 signaling [70]
OTULIN	Met-1 [71,72,73]	Prevents inflammation [71,72,73]
	STAT1 [74]	Controls antiviral signaling [74]
OTUB1	TRAF3/TRAF6 [75]	Negatively regulates virus-triggered type I IFN induction [75]
	UBC13 [76]	Augments NF-κB-dependent Immune Responses [76]
	AKT [77]	Controls the activation of CD8 + T Cells and NK Cells [77]
	RhoA [78]	Increases bacteria uptake [78]
	RIG-1 [79]	Activates RIG-I signaling cascade and antiviral responses [79]
	Smad2/3 [80]	Enhances TGFβ signaling [80]
OTUD1	MAVS/TRAF3/TRAF6 [81]	Inhibits Innate Immune Responses [81]
	IRF3 [82,83]	Maintains immune homeostasis [82] Negatively regulates Type I IFN induction [83]
OTUD4	MyD88 [84]	Suppresses TLR signaling [84]
	MAVS [85]	Regulates innate antiviral responses [85]
	ALKBH3 [86]	Regulates DNA damage [86]
A20	TRAF6 [87]	Restricts TLR signals [87]
	TRAF2/TRAF6/Ubc13/UbcH5c [88]	Inhibits NF-kappa B Signaling [88]
MCPIP1	TRAF6 [89]	Impedes NF-κB and inflammatory signaling [89]
ATXN3	HDAC3 [90]	Positively regulates type I IFN antiviral response [90]
JOSD1	MCL [91]	Inhibits mitochondrial apoptotic signaling [91]
	SOCS1 [92]	Inhibits type I IFN signaling and antiviral response [92]
POH1	pro-IL-1β [93]	Negatively regulates the immune response [93]
BRCC3	NLRP3 [94]	Promotes the inflammasome activation [94]
STAMBP	NALP7 [95]	Reduces pro-inflammatory stress [95]

## Abbreviations

ABRO1	Abraxas Brother 1
ALI	Acute lung injury
ALKBH3	AlkB homologue 3
AMSH	Anti-Müllerian hormone
ARDS	Acute respiratory distress syndrome
ASC	Apeck-like protein containing a CARD
ATXN3	Ataxin3
BRISC	BRCC36 isopeptidase complex
CBP	CREB-binding protein
cIAP-1	Cellular inhibitor of apoptosis protein-1
CFTR	Cystic fibrosis transmembrane conductance regulator
CSN5	COP9 signalosome 5
COVID-19	Coronavirus disease 2019
CYLD	Cylindromatosis
DUBs	Deubiquitinating enzymes
E2F1	E2F transcription factor 1
EIF3	Eukaryotic translation initiation factor 3
HBO1	Histone acetyltransferase binding to origin recognition complex 1
HDAC2	Histone deacetylase 2
IFN	Interferon
IKKγ	IκB kinase γ
IL-1β	Interlukin-1β
IRF3	Interferon regulatory factor 3
JAMMs	Zn-JAB1/MPN/MOV34 domain metallopeptidase
LPA1	Lysophosphatidic acid receptor 1
LPS	Lipopolysaccharide
LUBAC	Linear ubiquitin chain assembly complex
MAVS	Mitochondria antiviral-signaling protein
MCL1	Myeloid cell leukemia 1
MINDYs	Motif interacting with ubiquitin - containing novel DUB family
MJDs	Machado-Josephin disease protein domain protease
NEMO	Nuclear factor (NF)-κB essential modulator
NALP7	NACHT, LRR and PYD domains-containing protein 7
NFAT	Nuclear factor of activated T cells
NICD1	NOTCH1 intracellular domain
NLRP3	NLR family pyrin domain containing 3
MYSM1	MPN domain containing (MPND, myb-like SWIRM and MPN domains 1
OTUs	Ovarian tumor proteases
PAI-1	Plasminogen activator inhibitor-1
PEG10	Paternally expressed gene 10
PLY	Pneumolysin
POH1	Proteasome non-ATPase regulatory subunit 14
PRPF8	Pre-mRNA-processing-splicing factor 8
PSMD7	Proteasome non-ATPase regulatory subunit 7
RIG-1	Retinoic acid-inducible gene I
RIPK1	Receptor-interacting protein kinase 1
SARS-CoV-2	Severe acute respiratory syndrome coronavirus 2
STAMBP	STAM-binding protein
STING	Stimulator of interferon
TAK1	TGF-β-activated kinase 1
TGFβ-1	Transforming growth factor β-1
TRIF	TIR domain-containing adaptor inducing interferon-β
TNF-α	Tumor necrosis factor-α
TRAF	Tumor necrosis factor receptor-associated factor
UBA	Ub-activating enzymes
UBC	Ub-conjugating enzymes
UCHs	Ubiquitin carboxy-terminal hydrolases
USPs	Ubiquitin-specific proteases
